# Diversity of *Trichinella* species in carnivores from Bosnia and Herzegovina

**DOI:** 10.1186/s12917-024-03964-6

**Published:** 2024-03-23

**Authors:** Jasmin Omeragić, Naida Kapo, Vedad Škapur, Ćazim Crnkić, Šejla Goletić, Adis Softić, Teufik Goletić

**Affiliations:** 1 University of Sarajevo – Veterinary Faculty, Zmaja od Bosne 90, Sarajevo, 71 000 Bosnia and Herzegovina; 2 University of Sarajevo - Faculty of Agriculture and Food Science, Zmaja od Bosne 8, Sarajevo, 71 000 Bosnia and Herzegovina

**Keywords:** Bosnia and Herzegovina, *Trichinella* species, Carnivores, Zoonotic

## Abstract

**Background:**

In Bosnia and Herzegovina, domestic and wild carnivores represent a significant driver for the transmission and ecology of zoonotic pathogens, especially those of parasitic aetiology. Nevertheless, there is no systematic research of *Trichinella* species in animals that have been conducted in Bosnia and Herzegovina, even though trichinellosis is considered the most important parasitic zoonosis. The available results of the few studies carried out in Bosnia and Herzegovina are mainly related to the confirmation of parasitic larvae in the musculature of domestic pigs and wild boars or data related to trichinellosis in humans. The objective of our study was to present the findings of a comprehensive investigation into the species composition of *Trichinella* among 11 carnivorous species within the territory of Bosnia and Herzegovina, as follows: red fox (*Vulpes vulpes*), grey wolf (*Canis lupus*), brown bear (*Ursus arctos*), wildcat (*Felis silvestris*), pine marten (*Martes martes*), European badger (*Meles meles*), weasel (*Mustela nivalis*), European polecat (*Mustela putorius*), Eurasian lynx (*Lynx lynx*), but also dog (*Canis lupus familiaris*) and cat (*Felis catus*).

**Results:**

In the period 2013–2023, carnivore musculature samples (*n* = 629), each consisting of 10 g of muscle tissue, were taken post-mortem and individually examined using the artificial digestion method. In the positive samples (*n* = 128), molecular genotyping and identification of parasitic larvae of *Trichinella* spp. were performed using a PCR-based technique up to the species/genotype level. Positive samples were used for basic PCR detection of the genus *Trichinella* (rrnS rt-PCR technique) and genotyping (rrnl-EVS rt-PCR technique). The *Trichinella* infection was documented for the first time in Bosnia and Herzegovina among red foxes, grey wolves, brown bears, dogs, badgers and Eurasian lynx, with a frequency rate of 20.3%. Additionally, the presence of *T. britovi* infection was newly confirmed in Bosnia and Herzegovina, marking the initial documented cases. Furthermore, both *T. britovi* and *T. pseudospiralis* infections were observed in the wildcat population, whereas *T. britovi* and *T. spiralis* infections were detected in pine martens. Consistent with previous research, our findings align particularly regarding carnivores, with data from other countries such as Germany, Finland, Romania, Poland and Spain, where *T. britovi* exhibits a wider distribution (62.5–100%) compared to *T. spiralis* (0.0–37.5%). *T. britovi* is more common among sylvatic carnivores (89.0%), while *T. spiralis* prevails in wild boars (62.0%), domestic swine (82.0%) and rodents (75.0%).

**Conclusion:**

The results of our study represent the first molecular identification of species of the genus *Trichinella* in Bosnia and Herzegovina. Additionally, our findings underscore the necessity for targeted epidemiological studies to thoroughly assess trichinellosis prevalence across diverse animal populations. Considering the relatively high frequency of trichinellosis infection in investigated animal species and its public health implications, there is an evident need for establishing an effective trichinellosis surveillance system in Bosnia and Herzegovina.

## Background

Despite the undeniable impact of domestic and wild carnivores on the ecology and transmission of zoonotic pathogens and parasites, this area has been very poorly investigated in Bosnia and Herzegovina (B&H). In addition to wild carnivores’ diseases of special interest for public health such as rabies, tularemia, brucellosis, echinococcosis, toxoplasmosis and scabies, one should also take into account the public health importance of trichinellosis. Trichinellosis is a globally widespread parasitic disease that is transmitted by eating raw or semi-raw meat infected with *Trichinella* larvae [1]. Parasites from the genus *Trichinella* have an indirect development and life cycle, and the infected individual represents both the definitive and transitional host for this parasite. Currently, it has been described 10 species and 3 genotypes of the genus *Trichinella*, further divided into two clades: encapsulated (*Trichinella spiralis*, *T. nativa*, *T. britovi*, *T. nelsoni*, *T. murelli* and *T. patagoniensis*, *T. chanchalensis* - T6, T8 and T9) and non-encapsulated species (*T. pseudospiralis*, *T. papuae*, *T. zimbawensis*) [2, 3]. The main reservoirs and definitive/transitional hosts of these parasites are carnivorous and omnivorous mammals, birds and reptiles, distributed on all continents except Antarctica [2, 4]. Despite the indisputable impact of man on the environment and, consequently changing the ecosystem, this parasite resists and is the most represented in wild, domestic animals and humans in Eastern Europe, Asia and South America [1].

In Europe, the red fox (*Vulpes vulpes*) and the raccoon dog are considered the primary reservoir hosts for *Trichinella* [5]. Mustelids (such as badgers, martens etc.) and other carnivores (brown bears, lynxes, grey wolves etc.) may also serve as sources of infection [5]. In the Balkan region where *Trichinella* infections are endemic in domestic pigs, *T. spiralis* and *T. britovi* are the two most common species detected in both domestic and wild animals in Serbia, Croatia, Hungary, Romania, Bulgaria and North Macedonia [6]. In B&H, human and animal trichinellosis represents a significant public health problem and is the most prevalent food-borne parasitic zoonosis. In the last 20 years, there have been recorded more than 2,000 human cases of trichinellosis, even with a fatal outcome [7]. Similarly, the reports from Western Balkans indicate a high annual incidence of human trichinellosis. The number of patients per 100,000 inhabitants in Serbia is estimated to be 5.0 cases, 4.1 cases in B&H, 2.9 to 8.5 cases in Romania, 1.7–4.8 cases in Croatia, and 2.4 to 2.9 cases in Bulgaria [8].

Systematically organized identification of *Trichinella* species using PCR-based molecular techniques was not carried out in B&H ever before. The available results of the few studies are mainly related to the findings of parasitic larvae in the musculature of domestic pigs and wildlife (mostly wild boars), or data related to human trichinellosis. The first molecular identification of *T. pseudospirals* and *T. spiralis* from isolated parasitic larvae originating from domestic pigs, and *T. britovi* from isolated parasitic larvae originating from wild boar in samples originating from B&H, was carried out at the Serbian National Reference Laboratory for Trichinellosis, Institute for the Application of Nuclear Energy (INEP) in 2016 [9].

Considering the scarcity of scientific data on the presence of *Trichinella* species in animal populations in B&H, the public health importance of *Trichinella* and substantial wildlife biodiversity, this research has been conceptualized at the University of Sarajevo – Veterinary Faculty. This study aimed to determine the *Trichinella* species and identify the genotypes of the *Trichinella* species originating from different species of wild and domestic carnivores from the territory of B&H.

## Results

*Trichinella* spp. larvae were determined in 128 of 629 (20.3%) muscle samples, in a total of 11 animal species, namely: red fox (*Vulpes vulpes*), grey wolf (*Canis lupus*), brown bear (*Ursus arctos*), wildcat (*Felis silvestris*), marten (*Martes martes*), badgers (*Meles meles*), weasel (*Mustela nivalis*), European polecat (*Mustela putorius*), Eurasian lynx (*Lynx lynx*), but also in the dog (*Canis lupus familiaris*) and the cat (*Felis catus*). The detailed results showing the presence of *Trichinella* spp. in different host species, as well as descriptive statistical indicators of the intensity of larval infection (LPG) are shown in Table 1.

**Table 1:** Frequency (%) and intensity of infection (LPG) with *Trichinella* spp. larvae in animals in B&H

Hosts	n	n_pos_	%	LPG (min-max)	No. of positive/ investigated localities
**Felidae**					
Wildcat (*Felis silvestris*)	29	12	41.38^ab^	6.9 (0.4–25)	6/8
				0.1	
Eurasian lynx (*Lynx lynx*)	1	1	100^n/a^	0.61	1/1
Cat (*Felis catus*)	8	0	0.0^c^	0	0/7
**Canidae**					
Grey wolf (*Canis lupus*)	36	14	38.89^ab^	6.9 (0.6–33)	6/11
Red fox (*Vulpes vulpes*)	475	73	15.37^c^	8.8 (0.1–64)	30/58
Dog (*Canis lupus familiaris*)	21	3	14.29^bc^	2.4 (0.6–5.5)	2/9
**Ursidae**					
Brown bear (*Ursus arctos*)	29	15	51.72^a^	6.2 (0.8–32)	9/14
**Mustelidae**					
Pine marten (*Martes martes*)	16	8	50.0^a^	6.0 (1.1–20,0)	3/7
				1.5	
Badger (*Meles meles*)	6	2	33.33^abc^	2.7 (1.2–4.2)	2/4
Weasel (*Mustela nivalis*)	2	0	0.0^np^	-	0/2
European polecat (*Mustela putorius*)	2	0	0.0^np^	-	0/2
**Total**	629	128	20.35	n/a	n/a

n_pos_ – number of animals infected with larvae of *Trichinella* spp.; LPG – mean value of the number of larvae of *Trichinella* spp. in 1 g of musculature (minimum and maximum number of larvae in 1 g); ^abc^ – frequency values in the same column that do not contain a common letter mark are significantly different at the *p* < 0.05 level; n/a – not applicable (when the number of tested samples is *n* < 5)

Specific molecular detection of parasitic DNA using multiplex PCR (rrnl -EVS rt-PCR) method was performed in all 128 *Trichinella* spp. positive samples (Table 2). *T. britovi* was identified in 126 (98.4%) samples, while *T. spiralis* (0.78%) and *T. pseudospiralis* (0.78%) were detected in one sample each.

**Table 2:** *Trichinella* spp. species identified by multiplex PCR (rrnl-EVS rt-PCR) technique

Hosts	n_pos_	Samples PCR^1^ Ct range	Determined Trichinella spp. ^2^
**Felidae**			
Wildcat (*Felis silvestris*)	12	18.2–34.3	*T. britovi* (11) *T. pseudospiralis* (1)
		19.7	
Eurasian lynx (*Lynx lynx*)	1	18.3	*T. britovi* (1)
**Canidae**			
Grey wolf (*Canis lupus*)	14	17.3–25.8	*T. britovi* (14)
Red fox (*Vulpes vulpes*)	73	17.9–34.2	*T. britovi* (73)
Dog (*Canis lupus familiaris*)	3	20.4–24.6	*T. britovi* (3)
**Ursidae**			
Brown bear (*Ursus arctos*)	15	19.7–29.4	*T. britovi* (15)
**Mustelidae**			
Pine marten (*Martes martes*)	8	19.3–29.7	*T. britovi* (7)
		26.4	*T. spiralis* (1)
Badger (*Meles meles*)	2	18.7–23.2	*T. britovi*

n_pos_ – number of animals infected with larvae of *Trichinella* spp.; ^1^- Positive controls (PCR – Ct range): *T. spiralis* (Ts) 14.5; *T. pseudospiralis* (Tps) 14.7; *T. britovi* (Tb) 16.0; *T. nativa* (Tb) 16.3; ^2^- the number of animals in which a given species of *Trichinella* was identified.

## Discussion

Ecological diversity provides various conditions for the development and spread of *Trichinella* and may influence the frequency and distribution of this parasitic infection in wild carnivores. With 53% forest cover in B&H, crucial habitat for many wildlife species, studying *Trichinella* presence provides insights into parasite ecology and human health risks, especially in areas with high biological diversity such as B&H. The identification of *Trichinella s*pp. in several carnivore species from all parts of B&H represents the result of paramount importance in understanding the epidemiology of trichinellosis in B&H. The main finding of our study is reflected in the first identification of *T. britovi* in the populations of wild carnivores such as red foxes, grey wolves, brown bears, badgers, wildcats, pine martens and Eurasian lynx, but also domestic dogs. Further, there is a first identification of *T*. *pseudospiralis* in samples originating from wildcats, and *T*. *spiralis* in pine martens. The distribution and frequency of *Trichinella* spp. among the examined animal species were unequal and statistically significant (*p* < 0.001). Except for the Eurasian lynx, (only one animal positive for *T. britovi*), *Trichinella* spp. were most commonly represented in the population of brown bears. This finding was not significantly different in comparison to the frequency of *Trichinella* spp. in wildcats, grey wolves, pine martens and badgers, but it was significantly different in comparison to red foxes and domestic carnivores (dogs and cats). Despite these observed differences in frequency, it is important to consider the limitation stemming from the smaller sample size for some of the examined species, which could affect the interpretation of the established frequency results. The intensity of the infection, expressed through the number of *Trichinella* larvae per gram of sample (LPG), is a very important indicator for determining the possibility of infection and clinical manifestations of the disease. The results of our study are similar to those obtained in the study conducted by Pozio [10], who pointed out that the intensity of infection with *T. britovi* (average LPG) was higher in comparison with the infection with *T. spiralis*. The possible explanation for this fact can be found in the characteristics of the *Trichinella* species and differences in animal habitat. Wild carnivores (the main hosts of *T. britovi)* are more represented in areas with higher altitudes (colder climate) compared to domestic pigs and wild boars (the main hosts of *T. spiralis*). *T. britovi* is more resistant to freezing temperatures, it can survive up to 11 months in the muscle tissue of carnivores at -15ºC, and in the muscle tissue of pigs at -20ºC for up to three weeks, while the *T. spiralis* larvae at the same temperatures survive for a few days to a few weeks at most [10]. This is supported by the data provided by Blaga et al. [11] for Romania. Searching for 40 isolates, *T. britovi* (*n* = 26; 65.0%) was more prevalent and was predominantly found in wild animals (24/28; 86.0%). However, research in Serbia revealed that *T. spiralis* had a prevalence rate of 77.8% among wild carnivores, particularly red foxes and wildcats, while *T. britovi* exhibited a prevalence rate of 22.2% in the same hosts. The dominance of *T. spiralis* among wild animals in Serbia implies the transfer of this species from domestic to wild animals, which may be associated with numerous factors (natural features, forested areas, wild animals, agricultural areas, rural population preference for extensive pig breeding, education, public awareness etc.) [12].

## Felidae

### Wildcats

**Fig. 1:**
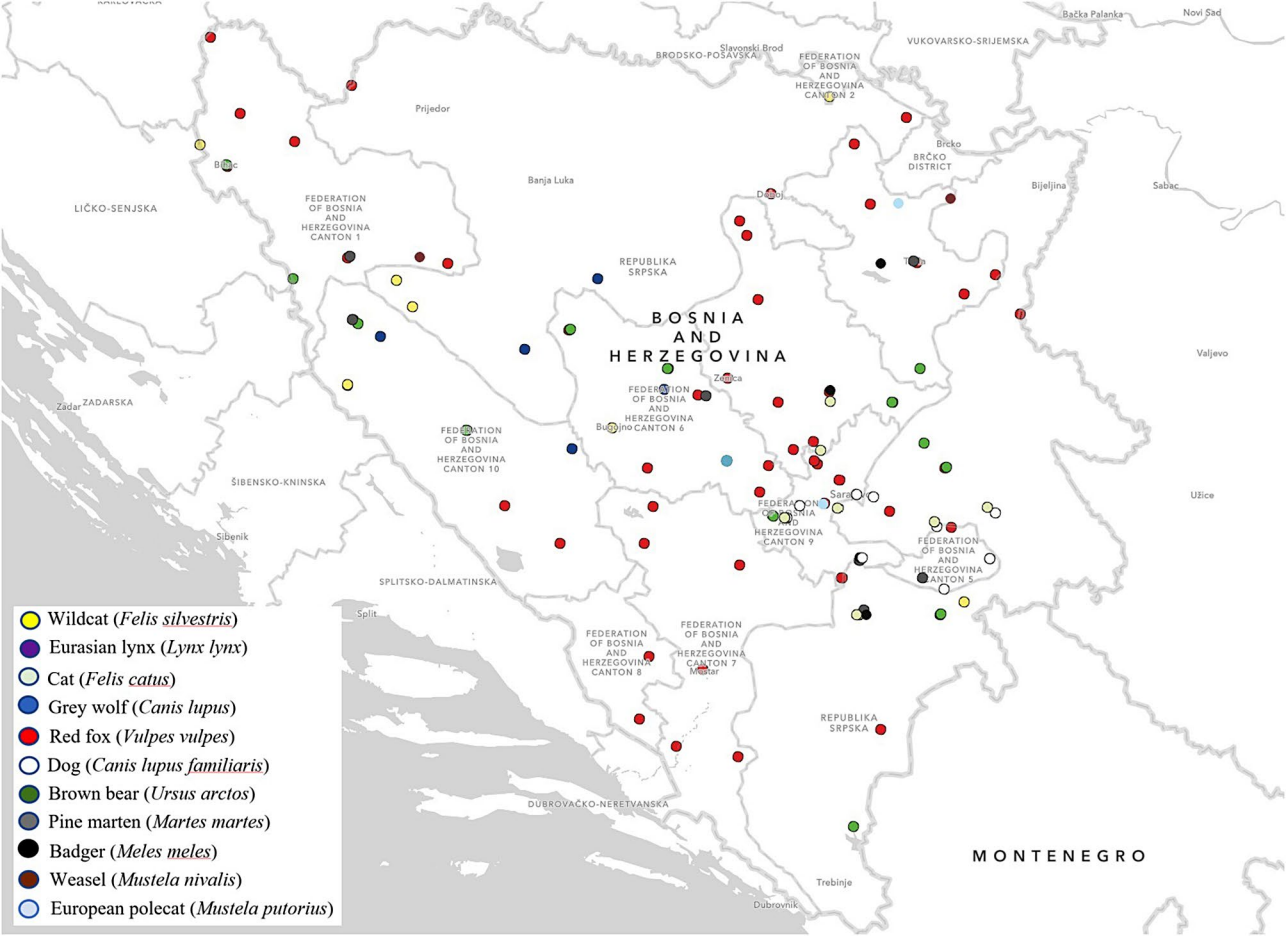
Map of Bosnia and Herzegovina illustrating the locations where positive *Trichinella* infections were identified. The study map was developed using ArcGIS® software (ESRI, Redlands, CA, USA), version 3.2

Our study results showed that 12 (41.3%) of investigated muscle samples originating from wildcats (*Felis silvestris*) were found to be infected with *Trichinella* spp., with an average LPG of 6.4. Of these, *T. britovi* larvae were identified in 11 samples, while *T. pseudospiralis* was identified in only one sample. Data on trichinellosis in wildcats in the Balkans are scarce, i.e., available in a few studies only. Thus, Brglez [13] estimated that the prevalence of trichinellosis in wildcats was 4.5%, while Cvetković et al. [14] highlighted the finding of *T. spiralis* in the population of wildcats in Serbia. The prevalence of *Trichinella* spp. in wildcats in Romania was 33.3% and 14.0%, respectively [11, 15]. Before our study, *T. pseudospiralis* was confirmed in only a sample originating from the domestic pig from Modriča within B&H [9]. In our study, we confirmed the presence of *T. pseudospiralis* and *T. britovi* in wildcats from the area of Bosanski Petrovac. Additionally, in other locations including Foča, Bihać, Trnovo, Kalinovik and Drvar, only the presence of *T. britovi* in wildcats was confirmed (Fig. 1). The number of localities where *Trichinella* spp. positive wildcats were found in comparison with the number of total investigated localities (6/8) was among the highest, but statistically similar to the ratios found in other host species. The representation of *Trichinella* spp. in the population of wildcats was significantly higher in comparison to the representation of this parasite in the population of cats and red foxes, but statistically insignificant in comparison to the other investigated animal species.

### Eurasian lynx

**Fig. 2:**
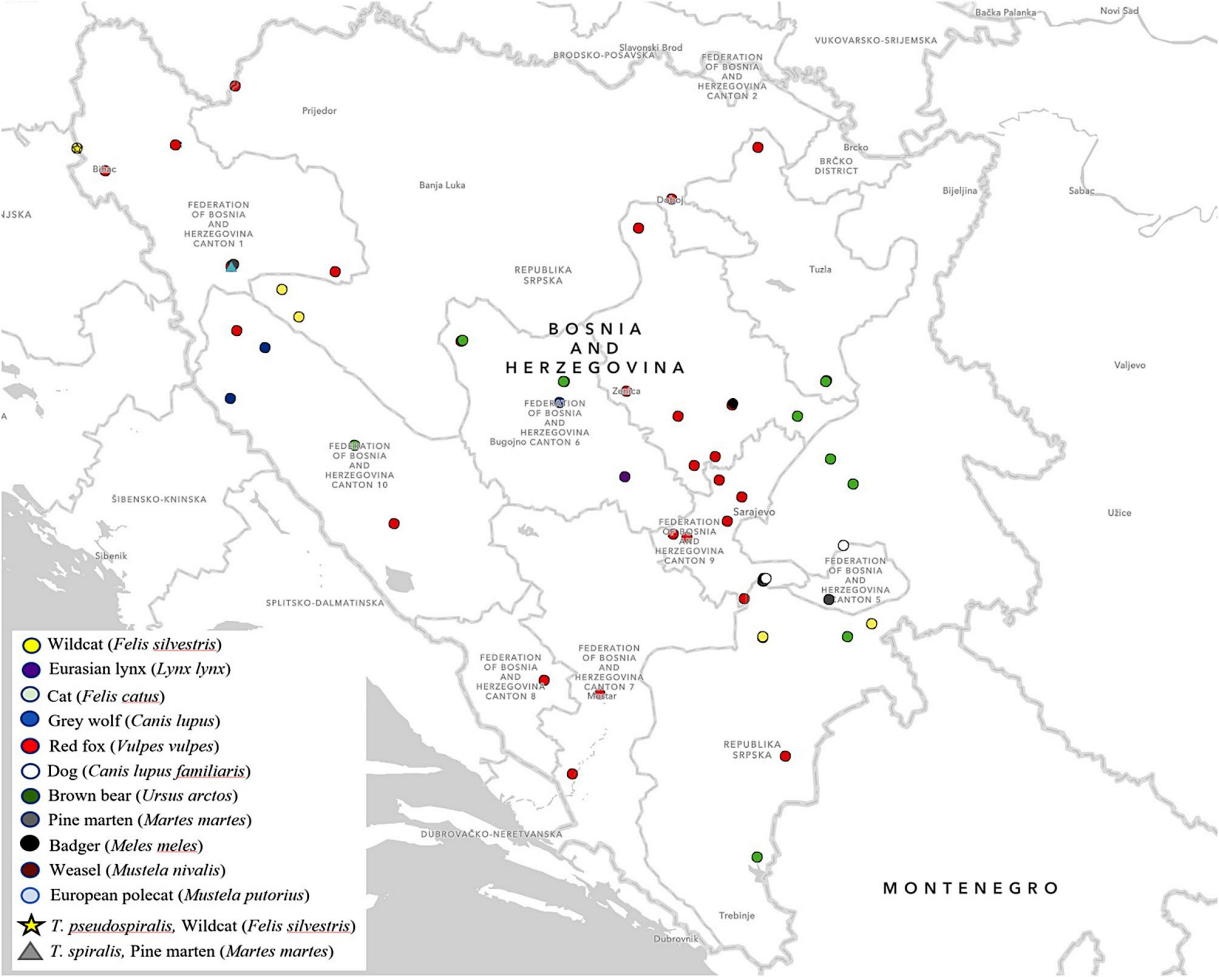
Map of Bosnia and Herzegovina with *Trichinella* investigated municipalities (67 in total). The study map was developed using ArcGIS® software (ESRI, Redlands, CA, USA), version 3.2

*T. britovi* was found in one sample originating from Eurasian lynx (*Lynx lynx*) with an average LPG of 0.61. The finding of *T. britovi* in the Eurasian lynx is the first finding of such kind in B&H. The positive sample originated from a Eurasian lynx in the Fojnica area (Fig. 2). Similarly, the *T. britovi* infection of Eurasian lynx have previously been described in other European countries such as Switzerland, Finland, Estonia, Slovakia and Latvia [13, 16–21].

### Cat (*Felis catus*)

None of the cat samples tested positive for the presence of *Trichinella* larvae. The cats originated from various locations within B&H (Fig. 2); however, the small number of samples is limiting in the context of discussing the true frequency of *Trichinella* infection in cats.

## Canidae

### Grey wolves

The relatively high prevalence of *T. britovi* in grey wolves (38.8%) in B&H partially supports the results of a previous study which reported that *T. britovi* is the main causative agent of sylvatic trichinellosis in areas of Europe and Asia with a moderate climate, and besides red foxes, martens, jackals and bears, grey wolves seem to be one of the main reservoirs of this parasite [8]. For the areas of the Balkans, Brglez [13] estimated the prevalence of *Trichinella* spp. in grey wolves at 44.0%, while in Croatia *T. britovi* was identified in all positive grey wolves samples with a previously established prevalence of 27.0% [22]. Similarly, all *Trichinella* spp. positive grey wolves samples (4/4, 100%) in Serbia were also identified as *T. britovi* [23]. The prevalence of grey wolf trichinellosis in Europe ranged between 8.9% in Italy [24], 31.0% [11] and 40.0% [15] in Romania, up to 97.5% in Russia [25]. In our study, *T. britovi* was confirmed in grey wolves from the locations Foča, Drvar, Bosansko Grahovo, Trnovo and Travnik, characterized by forested areas (Fig. 1). Determining *T. britovi* larvae in grey wolves did not differ significantly compared to localities where other infected animals were found, although the differences in percentages were very large. The highest frequency of infection was observed in grey wolves from the areas of Foča and Trnovo, while the lowest was in Drvar and Bosansko Grahovo. Concerning other species of animals, the frequency of trichinellosis in grey wolves was significantly higher in comparison to the prevalence of trichinellosis in red foxes and cats. Additionally, with grey wolves, an average LPG of 6.9 (range, 0.6–33) was noted. The range of LPG values, from low to high, was observed among grey wolves collected close to each other. Moreover, even among grey wolves from the same territory, there was a notable variability in LPG levels. Regarding other animal species, the higher frequency of trichinellosis in grey wolves has been found in other studies [26], where the reasons mentioned are related to their apex predator status and longer lifespan compared to foxes and cats. Several studies on *Trichinella* spp. among wild mesocarnivores in Europe have also reported a higher prevalence of *Trichinella* spp. in grey wolves and other large mesocarnivores (e.g., Eurasian lynx) compared to sympatric mesocarnivores [17, 27].

### Red foxes

Our finding of *T. britovi* in red foxes (15.3%) is supported by the results of other studies in Norway 4.8% [28]; Germany 0.08–0.2% [6]; Romania 7.0%, [11] and 21.5% [29]; Switzerland 1.6% [16]; Spain 0.3% [30]; Poland 5.7% [31] and 4.9% [32]; Serbia 6.7% [23]; Portugal 2.1% [33] and North Macedonia 21.5% [14]. According to available data, only Cvetkovic et al. [14] in the territory of Macedonia (21.5%) and Imre et al. [34] in Romania (21.5%) have reported a higher prevalence of red fox trichinellosis. The finding of only *T. britovi* in red foxes is consistent with studies in Belgium, Italy, Portugal, Switzerland and the Netherlands, while in Ireland and in the Swiss Alps area near the border with France, only the presence of *T. spirali*s has been confirmed [16, 23, 33]. Positive samples of red foxes have been confirmed at multiple locations in B&H; however, the highest frequency of infection was confirmed in the areas of Trnovo, Kladanj, Ilidža and Bosanska Krupa (Fig. 1). The representation of *T. britovi* in the population of red foxes was significantly lower than in bears, pine martens, wildcats and grey wolves, while no statistical differences were found in comparison to other animal species. This can be associated with the different number of samples from various carnivore species included in our study.

The mean value of the number of *Trichinella* spp. larvae found in the musculature of red foxes were 8.8 LPG, and the intensity of infection ranged from 0.1 LPG to 64.0 LPG. This result is consistent with previous studies. Thus, the average LPG for the infection in red foxes ranged from 0.12 LPG (0.02 LPG − 0.6 LPG) [35], 2.75 LPG to 4.4 LPG [36] in Poland, 2.4 LPG (0.3 LPG – 216 LPG) in Germany [35], 1 LPG to 15 LPG in North Macedonia [14], and 10.5 LPG in Romania [29].

### Dogs

The *Trichinella* spp. infection was confirmed in 14.2% of samples originating from stray dogs in only two of the nine investigated localities in B&H, specifically in the areas of Trnovo and Prača, characterized by forested areas (Fig. 1). The frequency of *T. britovi* in dogs was significantly lower than in bears and martens, but insignificant in comparison with other investigated animal species. Although the consumption of dog meat has been nominated as one of the common sources of human trichinellosis worldwide [6], this is not the problem in B&H and the consumption of dog meat cannot be considered a source of infection for humans due to cultural dietary habits. However, since stray dogs are primarily fed with human food leftovers, they can serve as sentinel animals.

Similarly to our findings, dog trichinellosis is present in neighbouring countries and Europe. Thus, 23.3% of investigated dog samples were found to be *Trichinella* spp. positive in Serbia, identifying *T. spiralis* in all positive samples [23]. In Kosovo, *Trichinella* spp. larvae were found in 16.0% of investigated dog samples, nominating *T. britovi* as the predominant parasitic species [37]. In Greece, *T. spiralis* was identified in 4.3% of investigated dog samples [38]. In Slovakia, *Trichinella* spp. was found in 12.8% of investigated dogs, and the vast majority of positive cases were distributed in rural areas in regions with the highest number of red foxes. The authors initiated the research due to the trichinellosis epidemic in Slovakia in 1998, associated with the consumption of smoked pork sausages to which trichinellosis dog meat was added [38].

## Ursidae

### Brown bears

The high prevalence of *T. britovi* in brown bears (51.7%) discovered in our study is consistent with the results of other studies of trichinellosis in animals in Europe [6, 11, 17, 35, 39, 40] but also in humans [41]. In ex-Yugoslavia, Brglez [13] stated that 16.8% of brown bears were infected with parasites from the genus *Trichinella*, and a study from Croatia reported 7.1% of infected brown bears [22]. Furthermore, among bears, an average LPG of 6.3 (range, 0.8–32) was observed. The highest frequency of *T. britovi* infection in brown bears was observed in the area of Kladanj, while the lowest was in the area of Foča (Fig. 1). The prevalence of trichinellosis in the population of brown bears was the highest in comparison to populations of all investigated animal species and significantly higher in comparison to the population of red foxes, dogs and cats. On the other hand, it was not significantly higher in comparison with the population of other investigated animal species.

The consumption of bear meat is not common or widespread among citizens in the area of B&H. However, illegal hunting and inadequate control of the trafficking and consumption of brown bear meat can have implications for public health. Furthermore, since the consumption of brown bear carrion can transmit *Trichinella* to other animals, it has been suggested that brown bears play a role as long-term reservoirs of *Trichinella* in the natural environment.

## Mustelidae

### Martens and badgers

*Trichinella* spp. were determined in 33.3% of samples originating from animals from the family Mustelidae. The most infected animal species was the pine marten with 50.0% *Trichinella* spp. positive samples. *T. britovi* was identified in 7 samples, and *T. spiralis* was identified in only one sample. In addition, *T. britovi* larvae were found in 33.3% of investigated badgers’ samples (33.3%). Our study results were similar to the results of other studies which indicated that *T. britovi* seem to be predominant *Trichinella* species in sylvatic carnivores such as martens (*Martes martes*) and badgers (*Meles meles*) [39, 40, 42]. On the contrary, Oivanen [17] and Pozio and Murell [39] estimated the frequency of *T. spiralis* in badgers (*Meles meles*). Pozio [6] and Gottstein et al. [41] pointed out that the clinical cases of human trichinellosis are most likely transmitted due to the consumption of badger and mink meat, which can represent a public health concern in certain countries. The finding of *T. britovi* in 33.3% of the investigated samples originating from badgers in our study is similar to the results of the study aimed at investigating the presence of *Trichinella* spp. in badgers in the Balkans, which reported 25.0% of *Trichinella* spp. positive badgers’ samples [13].

In the region of Bosanski Petrovac (Fig. 1), the finding of *T. spiralis* in a pine marten in our study represents the first record of such kind in the Balkan area. Similarly, the presence of *T. britovi* and *T. nativa* in pine martens in Europe is also an extraordinary finding and, to the best of authors’ knowledge, it is only recorded in Latvia and Lithuania [43–45]. The infrequent detection of *T. spiralis* in martens indicates that these animals likely have a minimal role in the maintenance or transmission of this parasite in the wild, consistent with earlier findings in various European countries [46, 47].

The mean larval burden in species from pine marten and badger (4.9 LPG in pine marten and 2.7 LPG in badger) found in the present study is comparable with the larval burden (6.8 LPG) detected in a previous study in Finland [40]. While it was notably higher (59.3 LPG) in pine martens tested in Poland [42]. The frequency of finding *Trichinella* spp. between these two species from the family Mustelidae was not statistically significant.

## Conclusion

Our research findings revealed that *Trichinella*, a causative agent of a parasitic disease, is widely distributed among wild mammals in our country. This indicates that the natural ecosystems of B&H are at risk of *Trichinella* invasion. Despite the relatively small sample size of animals under study, the results demonstrate the extensive presence of this parasite. Consequently, there is a genuine risk of human infection with *Trichinella* in areas where the animals were captured. Illegal hunting of wild animals such as brown bears and badgers is often the primary cause of human infections, where the meat of poached mammals is not subjected to veterinary and sanitary examination. This study is of utmost importance, as data on *Trichinella* prevalence in wildlife is very scarce in our region. In addition to the main finding, our study warrants the need for future specifically designed, epidemiologic studies on trichinellosis, which should investigate trichinellosis in areas and host species that were possibly underrepresented in the present study. Considering the relatively high prevalence of trichinellosis in investigated animal species and its public health implications, there is an evident need for establishing an effective trichinellosis surveillance system in B&H.

## Methods

### Study area

B&H is located in southeastern Europe, in the western part of the Balkan Peninsula. It is a mountainous country within the dominant mountain system of the Dinarides (Dinar Alps) on an area of 51,209.2 km^2^, with three basic climate zones: Mediterranean climate near the Adriatic Sea, continental mountainous in the central part of B&H and moderate continental in the northern and eastern parts. Forests and forest land cover 53.0% of the state territory. The percentage of forest cover has decreased by approximately 11.0–12.0% in the last 30 years. B&H is one of the countries in Europe with the greatest biodiversity of plant and animal species [48].

### Animal populations and sampling methods

In the period January 2015 - February 2023, the frequency of *Trichinella* spp. infection was investigated in 11 carnivore species (*n* = 629), as follows: the red fox (*Vulpes vulpes*), grey wolves (*Canis lupus*), brown bear (*Ursus arctos*), wildcat (*Felis silvestris*), pine marten (*Martes martes*), European badger (*Meles meles*), weasel (*Mustela nivalis*), European polecat (*Mustela putorius*), Eurasian lynx (*Lynx lynx*), but also a dog (*Canis lupus familiaris*) and a cat (*Felis catus*). The sampling procedure was conducted following the current legal norm [49], and in cooperation with veterinary and hunting organizations. All investigated carnivorous animal species were found deceased by hunting and veterinary organizations, except for red foxes, which were shot during an official survey on the effectiveness of rabies vaccination. None of these animals were killed for the purposes of our research. Additionally, samples were collected during necropsies of wild and domestic carnivores conducted at the University of Sarajevo - Veterinary Faculty to determine the cause of death, excluding samples from foxes. Muscle samples were collected from carcasses of the adult animals found in 67 areas (municipalities) across the country (Fig. 2). Musculature samples were taken from predilection sites (diaphragm root, tongue, masticatory muscles and tibial muscles). Samples were adequately labelled and kept at temperatures up to 4ºC until examination (no later than two days after admission). All samples were analyzed at the laboratory for parasitology of the University of Sarajevo - Veterinary faculty, accredited by BAS EN ISO/IEC 17025:2018.

### **Detection and identification of*Trichinella*spp. larvae**

#### Digestion method

All collected samples (*n* = 629) were tested for the presence of *Trichinella* spp. larvae, i.e., 10 g of musculature, with no connective and fatty tissue, were weighed and examined. All the collected muscle samples were individually examined by artificial digestion method according to the Commission Regulation (EC) no. 1375/2015 [50].

Larvae obtained by digestion of each sample were counted under a stereomicroscope (OLYMPUS, model CH20BIMF200) with a sub-stage transmitted light source of adjustable intensity, initially set at a magnification of 15 to 20x to ensure optimal examination of the samples. Magnification was then increased to 40-100x for detailed observation. For each sample, the number of larvae per g of sample (LPG) was determined to estimate the larval load. Collected larvae were washed in distilled water, preserved in 96% ethyl alcohol and stored in a freezer at -20 °C until DNA extraction and PCR analysis.

#### Extraction of nucleic acids

All molecular analyses of *Trichinella* spp. larvae were performed at the laboratory for molecular-genetic and forensic investigations of the University of Sarajevo - Veterinary faculty (BAS EN ISO/IEC 17025:2018). The DNA extraction was done from both the referent strain larvae as well as the sampled larvae of *Trichinella* spp. Before the extraction, each larva was homogenized by vortexing with two glass beads for 10 min. The extraction was performed using DNeasy Blood & Tissue Kit® (Qiagen, Hilden, Germany), according to the manufacturer’s instructions.

### **Detection of*Trichinella*genus by real-time qPCR**

The detection of a highly conserved mitochondrial small-subunit ribosomal RNA gene (rrnS) of the *Trichinella* genus was performed by an SYBR-Green Real-Time qPCR as described in Cuttel et al. [53]. Both primers were used at the final concentration of 300 nm and 5 µL of the extracted DNA was used for each reaction. The extracted DNA of a reference strain *T. britovi* was used as a positive control. The PCR reaction and melting curve analysis were done with Power SYBR® Green RNA-to-CT Kit (Applied Biosystems, USA), according to the manufacturer’s instructions, on a Stratagene Mx3005P® thermocycler (Agilent Technologies, USA). The thermoprofile consisted of polymerase activation at 90 °C for 10 min, 40 cycles of denaturation at 95 °C for 15 s, annealing at 58 °C for 30 s and extension at 72 °C for 30 s, one final extension at 72 °C for 10 min and a melting curve analysis in which the temperature was subsequently increased from 60 to 95 °C, with an increment of 0,5 °C/cycle. Fluorescence signals were measured and collected at the end of each extension step, and continuously during melting point analysis.

### **Genotyping of*Trichinella*species by multiplex real-time qPCR**

All samples that yielded positive results for *Trichinella* genus real-time qPCR were tested further to identify the species. Specifically, the multiplex qPCR used was to discriminate between species *T. spiralis*, *T. pseudospiralis*, *T. britovi* and *T. nativa* in a single run. The primers for discrimination between *T. spiralis*, *T. pseudospiralis* and *T. britovi* were as described in Guenther et al. [51], and for *T. nativa* were as described in a protocol by European Union Reference Laboratory for Parasites [52]. The amplification kit, thermoprofile and primer concentrations were the same as the ones described for the detection of the *Trichinella* genus. The results were interpreted by analysing the melting point temperatures of amplicons, which were specific for each tested species: 82 °C for *T. spiralis*, 78.3 °C for *T. pseudopiralis*, 82.8 °C for *T. britovi* and 76.5 °C for *T. nativa* [51–53].

### Spatial data processing and statistical analysis

The preparation, presentation, manipulation and analysis of spatial data (maps) was performed using GIS software MapInfo Professional version 9.0. Statistical analysis of the results was performed using Minitab 17 software [54]. Differences in the frequency of positive samples between animal species or between localities were analyzed using the chi-square test. The test showed a significant effect (*p*-value < 0.05) in the frequency of positive findings between animal species, and the differences between individual species were further tested by the chi-square test with Yates’ correction for continuity. Fisher’s exact test was used for comparisons of a small number of cases (*n* ≤ 50). *P*-values less than or equal to 0.05 were considered statistically significant.

## Data Availability

All data generated or analysed during this study are included in this article
